# Long-term outcomes of biodegradable versus 2nd generation durable polymer drug-eluting stents in PCI: Protocol for a systematic review and meta-analysis

**DOI:** 10.1371/journal.pone.0319946

**Published:** 2025-03-19

**Authors:** Rosa J Thuemmler, Trisha Choudhary, Yong Hui Tan, Maria-Bianca Andrei, Haipeng Liu, Niraj S Kumar

**Affiliations:** 1 Institute of Applied Health Sciences, School of Medicine, Medical Sciences and Nutrition, University of Aberdeen, Aberdeen United Kingdom; 2 National Medical Research Association, Leicester, United Kingdom; 3 Brighton and Sussex Medical School, University of Brighton and University of Sussex, Brighton, United Kingdom; 4 Bart’s and the London School of Medicine and Dentistry, London, United Kingdom; 5 Grigore T. Popa University of Medicine and Pharmacy, Iasi, Romania; 6 Centre for Intelligent Healthcare, Coventry University, Coventry, United Kingdom; 7 Department of Cardiovascular Sciences, University of Leicester, Leicester, United Kingdom; Shanghai Jiao Tong University Medical School Affiliated Ruijin Hospital, CHINA

## Abstract

**Background:**

More than 3 million individuals globally experience STEMI each year, with percutaneous coronary intervention (PCI) as the preferred revascularization method. While second-generation Drug Eluting Stents (DES) reduce restenosis compared to bare-metal stents, complications such as neoatherosclerosis and stent thrombosis remain. Second-generation stents, including durable polymer (DP-DES) and biodegradable polymer (BP-DES), aim to improve outcomes, though guidelines do not specify a preference. Given mixed results from prior studies and new long-term data, we aim to perform a systematic review and meta-analysis comparing long-term outcomes of DP-DES vs. BP-DES following PCI.

**Methods:**

This protocol has been developed following the Preferred Reporting Items for Systematic Review and Meta-Analysis Protocols. MEDLINE, Embase, and Scopus databases will be searched for eligible observational and interventional studies from inception up to 5^th^ of October 2024. Screening (title/abstract and full text), data extraction, risk of bias assessment, and quality of evidence assessment will be conducted by two independent reviewers. A random-effects model will be used to meta-analyse outcomes.

**Discussion:**

DES have greatly advanced PCI for STEMI. However, long-term stent thrombosis remains an issue due to chronic inflammation and impaired healing from the stent’s polymer coating. To overcome this, BP-DES were introduced to dissolve their coating within 2–9 months. However, whether BP-DES offers superior long-term outcomes compared to second-generation DP-DES remains uncertain. While previous meta-analyses have shown similar outcomes, recent studies suggest BP-DES may offer better long-term results. This review will compare long-term outcomes (≥5 years) of BP-DES vs. DP-DES, providing important insights to inform clinical practice.

**Systematic review registration:** PROSPERO (CRD42024592579)

## Background

ST-elevation myocardial infarction affects over 3 million individuals annually and contributes massively to global mortality rates [1]. Percutaneous Coronary Intervention (PCI) stands as the preferred reperfusion therapy for these patients [2,3].

Early PCI procedures restored blood flow to the myocardium via plain balloon angioplasty [3]. However, limitations such as abrupt vessel closure, dissections and vessel recoil limited the technique’s ability to maintain coronary vessel patency [4]. The introduction of stents have significantly improved post PCI outcomes, by overcoming these limitations [5].

Nevertheless, placing a stent into the artery presents its own challenges. Bare-metal stents can injure the vessel wall leading to neointimal hyperplasia (NIH) and restenosis [6]. Although, the advent of Drug Eluting Stents (DES) has notably reduced this risk, neoatherosclerosis and in-stent thrombosis still occur [6,7]. Subsequent target vessel revascularisation may be required as treatment, and is associated with a higher risk of adverse cardiac outcomes compared to PCI for de novo lesions [8]. As such, in-stent restenosis remains a public health burden [3].

Technological advances in stent manufacturing have led to the development of a newer generation of DES. Second-generation durable polymer DES (DP-DES) have a thin metal alloy platform of either cobalt-chromium or platinum-chromium coated with a biocompatible polymer and delivers novel antiproliferative drugs: Everolimus or Zotarolimus [9].This stent design intends to improve poor re-endothelialisation and reduce the thrombogenicity and inflammatory response associated with first-generation DP-DES [10]. Biodegradable polymer DES (BP-DES) are designed with a polymer coating that dissolves over a period of time, usually 2-9 months, whilst simultaneously delivering either Biolimus, Sirolimus or Everolimus [11]. Theoretically, these are thought to be better than DP-DES as the polymer that can trigger an inflammatory response is removed, reducing the risk of stent thrombosis [12]. Whilst previous meta-analyses comparing second-generation DP-DES to BP-DES exist, results remain inconclusive and demonstrate non-inferiority [13–17]. Moreover, while these studies have been based on randomised controlled trials (RCTs), several additional trials and large observational studies with longer-term follow-up data ( ≥ 5 years) have since been conducted. To the best of our knowledge, no comprehensive review has synthesised findings from these studies incorporating long-term outcomes, which have sparsely been reported on in the past. Assessing newly acquired data from RCTs and observational studies, particularly concerning long-term outcomes, is essential for guiding clinical practice in the future.

We aim to perform a systematic review and meta-analysis comparing short term as well as long-term outcomes following a PCI with second-generation DP-DES versus BP-DES. We aim to use both RCT and observational data, providing the most comprehensible review to date.

## Methods

### Design

**Fig 1:**
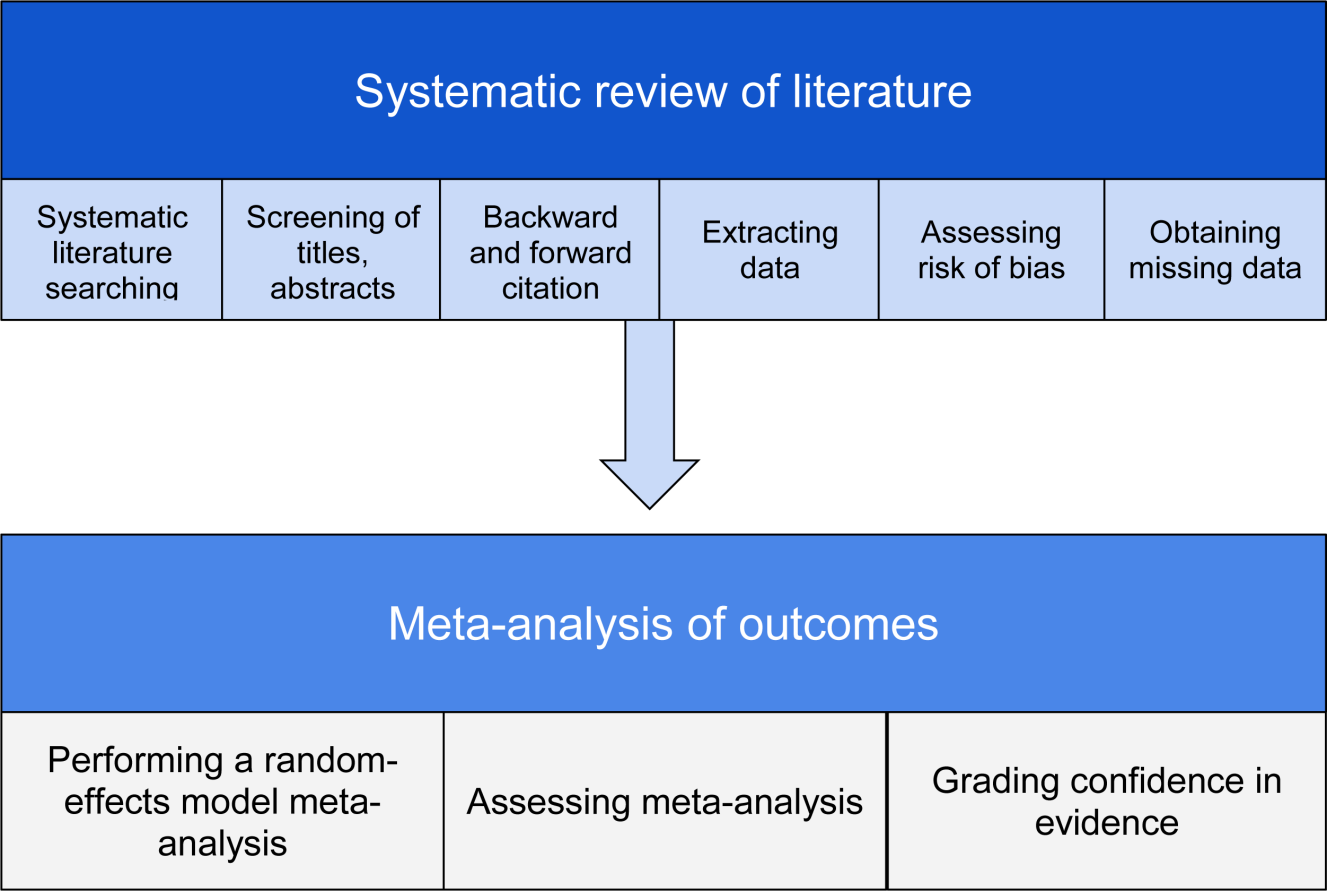
Overview of study design.

Our study protocol follows the Preferred Items for Systematic Reviews and Meta-analysis - protocol (PRISMA-P) checklist (available in Supplement S1) and has been registered on PROSPERO (CRD42024592579) [18]. The systematic review and meta-analysis will be reported in accordance with the PRISMA 2020 statement [19]. A summary of the study design is provided in Fig 1.

### Information sources

A comprehensive electronic search will be conducted in MEDLINE (Ovid), Embase (Ovid) and Scopus from inception up to the 5th of October 2024. Additional studies will be identified by manually searching the reference list of eligible full studies as well as bibliographies of relevant systematic reviews. Conference abstracts will not be included.

### Search strategy

Observational studies (i.e., cohort studies and registries) and interventional studies (i.e., randomised controlled studies) will be searched for long-term outcomes ( ≥ 5 years) after BP-DES versus DP-DES intervention. The search fields ‘Title’, ‘Abstract’, and ‘Medical Subject Headings (MeSH)/Thesaurus’ will be applied to ensure the best possible study retrieval. Detailed search strategies will be developed for each electronic database searched (Supplement S2)

### Eligibility criteria

We will include randomised control trials and observational studies conducted in the English language, reporting long-term outcomes ( ≥ 5 years) in patients following percutaneous intervention using BP-DES versus Second-Generation DP-DES. Details of the eligibility criteria are provided in Table 1.

**Table 1:** Details of the eligibility criteria used in this study in accordance with PICOS.

Patients	The population will consist of adults who have undergone percutaneous coronary intervention with either Biodegradable polymer drug eluting stents or Second-Generation durable polymer drug-eluting stent following a confirmed myocardial infarction
Intervention	Biodegradable polymer drug eluting stents
Comparator	Second-Generation durable polymer drug-eluting stent
Outcomes	The main review outcomes for this review are as follows: **Primary Outcomes** 1. Target lesion failure, defined as a composite of cardiac death, target vessel myocardial infarction or target lesion revascularisation. 2. Target vessel failure, defined as a composite of cardiac death, target vessel-related myocardial infarction, or clinically indicated target vessel revascularisation. **Secondary Outcomes** 1. All-cause death 2. Cardiac death 3. Stent thrombosis 4. Myocardial Infarction 5. Any revascularisation (defined as revascularisation involving either the target or non-target vessels) 6. Major adverse cardiac events (defined as all-cause death, any myocardial infarction, or clinically indicated target lesion revascularisation)
Study types	Randomised control studies and observational studies (e.g., cohort studies)

### Study selection

Studies will be independently selected by two reviewers (TC and RJT) by screening titles and abstracts in Rayyan systematic review management program. Studies identified as relevant will be subjected to full text screening in accordance with prespecified selection criteria. Reasons for exclusion will be documented. Reference lists of the included studies will be hand screened for potential studies. Any discrepancies at either stage will be adjudicated by a third author (NSK). The process of study selection will be summarised using a PRISMA flow diagram.

### Data collection and data management

Two individual reviewers (YT and MA) will extract and collect data on outcomes. A predefined extraction form will be developed and utilised by both reviewers. Data extraction will be undertaken from the peer reviewed published report and supplemented with data from clinical trials registry only if data is unavailable from publication. Disagreement will be resolved by a third reviewer (NSK). Depending on the data reported in the studies, we will collect the raw data, the aggregated outcomes or both. In studies reporting aggregated data, the estimated (i.e., mean and medians) will be extracted along with their variation (i.e., 95% confidence intervals, standard deviation, or range).

Data on study characteristics, participants characteristics, and intervention will be extracted into four predefined Excel sheets (Microsoft Corporation, Version 16.72, Redmond, WA) [20]. Sheet one will include all relevant study characteristics (author, year of publication, journal, study type, randomisation, blinding, location, inclusion criteria, exclusion criteria, follow-up), sheet two will include all population characteristics (age, sex, BMI, ethnicity (caucasian, black, asian, other), diabetes, hypertension, smoking status, hyperlipidaemia, malignancy, ESRD, vessel status disease (total occlusion, multivessel occlusion), previous cardiovascular events, previous PCI), sheet three will include intervention details (stent type (including strut thickness, drug, dose and brand) DAPT treatment and duration, Polymer biodegradation), sheet four will contain outcomes (Target lesion failure, target vessel failure, all-cause death, cardiac death, stent thrombosis, myocardial infarction).

### Missing data

Where possible missing values will be calculated from the available data (p-values, t-values, confidence intervals or standard errors). If data is not provided by the publication or registry, they will be annotated as not available. Where possible, attempts will be made to contact corresponding authors of relevant studies to obtain raw data.

### Data synthesis

Meta-analysis will be performed on all outcomes if the outcome variables are sufficiently homogenous and there is adequate sample size. Outcome variables that are not homogenous will be synthesised narratively. Statistical analyses will be performed using Review Manager (RevMan) 5.4.1.

The odds ratio (OR) will be used to analyse the dichotomous safety and efficacy outcomes. The uncertainty will be expressed with 95% confidence intervals (95% CI). Heterogeneity will be assessed by the chi-squared test on Cochrane’s Q statistic and I². I² values falling between 0% and 25% will be classified as insignificant heterogeneity, 25%-50% as low, 50%-75% as moderate and more than 75% as high heterogeneity [21]. A random effect model with the Mantel-Haenszel method or a fixed effect model will be used accordingly. τ² (tau-squared) will be estimated to determine the variance of true effect sizes across studies in a random-effects model. A p-value ≤ 0.05 is considered statistically significant. Subgroup analyses will be conducted, should sufficient data be available, for patients with diabetes mellitus. Sensitivity analyses will be carried out by omitting observational studies.

### Meta-bias

To assess meta-bias, outcome reporting will be evaluated by comparing the outcomes specified in the study protocol or methods section with those that are actually reported in the results. Publications bias will be visually assessed using a funnel plot. In the presence of publication bias, the funnel plot should resemble an asymmetrical funnel. Egger’s test will be conducted for outcomes reported by more than 10 studies.

### Risk of bias

The Risk of bias will be assessed using The Cochrane Risk of Bias Tool 2.0 for randomised control trials [22]. The following domains will be independently assessed: randomisation process, deviations from intended interventions, missing outcome data, measurement of the outcomes, and selection of the reported results. The ROBINS-I tool will be applied to non-randomised studies of intervention [23]. For both, studies will independently be assessed by two reviewers (RJT and TC). A third reviewer (NSK) will adjudicate, in case of disagreement.

### Confidence in evidence

Certainty of evidence will be assessed using the Grading of Recommendations, Assessment, Development and Evaluations (GRADE) approach where possible. Two reviewers (YT and MA) will independently score five categories of reasons for rating down the quality of evidence, and three categories for rating up, with a yes/no decision regarding rating up or down of each outcome. This will be conducted in accordance with a prespecified proforma (Supplement S3). Observation studies will start at a low quality of evidence and can be upgraded accordingly. GRADE results will be used to inform conclusions on the strength of outcomes in studies comparing Biodegradable polymer drug eluting stents to Second Generation DP-DES in patients following myocardial infarction. Studies will independently be assessed by two reviewers (RJT and TC). A third reviewer (NSK) will adjudicate, in case of disagreement.

## Discussion

In patients with acute myocardial infarction, the use of Percutaneous Coronary Intervention is considered the gold standard of treatment [2,3]. While drug-eluting stents (DES) have significantly reduced the need for repeat revascularisation compared to bare-metal stents, concerns persist regarding the long-term safety of the durable polymer coatings used in DES^.^ [6,7]. Second-generation DP-DES have maintained the low restenosis rates of ﬁrst-generation devices with reduced rates of stent thrombosis. However, very late stent thrombosis and neo-atherosclerosis, resulting in adverse clinical outcomes, have been observed with second-generation DP-DES [7]. This is believed to be due to an association with chronic inflammation and delayed endothelial healing, caused by the persistence of a durable polymer [7,24]. DES gradually releases drugs from polymer coatings, which prolongs the time of complete endothelialisation, resulting in thrombogenic sites [25]. This ongoing inflammatory response may substantially increase the risk of adverse outcomes, particularly in high-risk patients or those who discontinue antiplatelet therapy, as these inadequately healed areas become more susceptible to thrombosis [25].

To mitigate associated risks, BP-DES have been developed, reducing the potential for long-term issues related to polymer persistence in the coronary artery wall. Nonetheless, evidence for the superiority of one polymer strategy over another is still lacking. Previous meta-analyses and systematic reviews have reported clinically comparable long-term outcomes [12,26]. These studies, however, are limited by small scale RCTs and often lack specific data regarding more contemporary, second-generation DP-DES.

Recently, large-scale observational and RCT studies have emerged, demonstrating conflicting findings [27–30]. Studies have identified better clinical long-term outcomes in BP-DES compared to second-generation DES, including the BIOSTEMI trial, published in The Lancet, which reports the superiority of BP-DES concerning the long-term incidence of target lesion failure at 5 years [28]. In contrast, an observational study involving 7712 patients found an increased five-year risk of MACE and total revascularisation in BP-DES compared to DP-DES [29]. Notably, differences often become apparent only after 5 years of implantation, underscoring the need for further studies assessing long-term outcomes [28,29]. Furthermore, a large scale meta-analysis has identified diabetes mellitus as an independent risk factor associated with long-term adverse cardiovascular outcomes following PCI with BP-DES [31]. Subgroup analysis in this at-risk population is warranted and has been lacking in previous reviews.

There are limitations to this study protocol. Firstly, we acknowledge that combining observational studies and randomised control studies may lead to greater heterogeneity in the pooled results, making it more challenging to draw definitive conclusions. To address this, we will conduct a sensitivity analysis, to see if study design leads to differences in outcomes. Long-term follow-up data may involve older versions of BP-DES and DP-DES, which could limit the relevance of our findings to current PCI practices using more advanced stent technologies. Furthermore, the exclusion of unpublished literature and literature not written in English might affect the overall findings. Lastly, this meta-analysis includes studies from a variety of countries and ethnic groups. This heterogeneity may introduce variability in outcomes due to cultural, socioeconomic, and environmental contexts, which may limit generalizability.

The results of this review will provide the most up-to-date comparison of BP-DES and DP-DES with long-term follow-up. This is vital to determine whether a clinical benefit exists in BP-DES over second-generation DP-DES, particularly regarding long-term outcomes, where evidence has been scant. Exploring patient-specific factors, such as DM, may provide more insight into optimising stent selection, improving outcomes, and more personalised treatment strategies in patients undergoing PCI.

## Supporting Information

S1 File PRISMA-P Checklist.
(DOCX)

S2 File Strategy for database searches.
(DOCX)

S3 File Proforma used for GRADE assessment.
(DOCX)
