# Supplementary material for: Long-term outcomes of biodegradable versus 2nd generation durable polymer drug-eluting stents in PCI: Protocol for a systematic review and meta-analysis
Source: PLoS One. 2025 Mar 19;20(3):e0319946. doi: 10.1371/journal.pone.0319946 (PMC11922205; doi:10.1371/journal.pone.0319946)
Supplement: S2 File — Strategy for database searches. (DOCX) [file pone.0319946.s002.docx]

Objective: **To compare the long term outcomes of biodegradable polymer drug eluting stent (BP-DES) and 2nd generation durable polymer drug eluting stent (DP-DES) for percutaneous coronary intervention (PCI)**

**PICO Framework**

| Framework | Research |
| --- | --- |
| Population | Patients receiving percutaneous coronary intervention (PCI) |
| Intervention | Biodegradable Polymer Drug-Eluting Stents (BP-DES) |
| Comparison | 2nd Generation Durable Polymer Drug-Eluting Stents (DP-DES) |
| Outcome | Long-term results of target lesion failure, target vessel failure, all-cause death, cardiac death, stent thrombosis, myocardial infarction, any revascularisation, major adverse cardiac events, |

**Embase**

Search: 30/09/2024

|  | Search | Result |
| --- | --- | --- |
| #1 | 'long term effect*' OR 'clinical outcome'/exp OR 'clinical outcome' OR 'long term efficacy'/exp OR 'long term efficacy' OR 'treatment outcome'/exp OR 'treatment outcome' OR 'outcome assessment'/exp OR 'outcome assessment' OR 'follow up'/exp OR 'follow up' OR 'prognosis'/exp OR prognosis OR ‘lasting effect’ OR 'sustain* outcome*' OR 'prolong* consequences' OR 'future impact' OR 'long range result*' OR 'final outcome*' | 5881323 |
| #2 | 'biodegradable polymer' OR 'bioabsorbable polymer' OR 'bioresorbable polymer' OR 'resorbable polymer' OR 'absorbable polymer' OR 'degradable polymer' OR 'biodegradable stent' OR 'bioabsorbable stent' OR 'bioresorbable stent' OR 'resorbable stent' OR 'absorbable stent' OR 'degradable stent' OR 'biodegradable scaffold' OR 'bioabsorbable scaffold' OR 'bioresorbable scaffold' OR 'resorbable scaffold' OR 'absorbable scaffold' OR 'degradable scaffold' OR 'bp des' OR 'umirolimus'/exp OR umirolimus | 12434 |
| #3 | 'permanent polymer' OR 'durable polymer' OR 'long*lasting polymer' OR 'enduring polymer' OR 'stable polymer' OR 'persistent polymer' OR 'permanent stent' OR 'durable stent' OR 'long*lasting stent' OR 'enduring stent' OR 'stable stent' OR 'persistent stent' OR '2nd generation drug eluting stent' OR 'second generation drug eluting stent' OR 'second generation pharmaceutical*' OR '2nd generation pharmaceutical*' OR 'second generation therap*' OR '2nd generation therap*' OR 'dp des' OR 'zotarolimus'/exp OR zotarolimus OR 'zotarolimus eluting coronary stent'/exp OR 'zotarolimus eluting coronary stent' OR 'zotarolimus eluting stent'/exp OR 'zotarolimus eluting stent' OR 'everolimus'/exp OR everolimus OR 'everolimus eluting coronary stent'/exp OR 'everolimus eluting coronary stent' OR 'everolimus eluting stent'/exp OR 'everolimus eluting stent' OR 'novolimus'/exp OR novolimus OR 'novolimus eluting coronary stent'/exp OR 'novolimus eluting coronary stent' | 42761 |
| #4 | 'percutaneous coronary intervention'/exp OR 'percutaneous coronary intervention' OR 'percutaneous transluminal angioplasty'/exp OR 'percutaneous transluminal angioplasty' OR pci OR 'transluminal coronary angioplasty'/exp OR 'transluminal coronary angioplasty' OR 'angioplasty'/exp OR angioplasty OR 'coronary stenting'/exp OR 'coronary stenting' OR 'ptca catheter'/exp OR 'ptca catheter' OR 'percutaneous coronary angioplasty'/exp OR 'percutaneous coronary angioplasty' OR 'interventional cardiology procedure' OR 'percutaneous coronary procedure' | 253261 |
| #5 | #1 AND #2 AND #3 AND #4 | 1359 |
| #6 | #5 AND 'article'/it NOT 'case report'/de | 722 |

**Ovid Medline**

Search: 10/05/2024

Sources: Journals@Ovid Full Text <October 04, 2024>, Your Journals@Ovid, Ovid MEDLINE(R) and Epub Ahead of Print, In-Process, In-Data-Review & Other Non-Indexed Citations, Daily and Versions <1946 to October 04, 2024>

|  | Search | Result |
| --- | --- | --- |
| #1 | Prognosis.sh. or prognosis.af. or 'Long*term effect*'.af. or 'Clinical outcome*'.af. or 'Long*term efficacy'.af. or 'Patient outcome*'.af. or 'Follow*up outcome*'.af. or 'Lasting effect*'.af. or 'Sustain* outcome*'.af. or 'Prolonged consequences'.af. or 'Future impact'.af. or 'Long*range result*'.af. or 'Final outcome*'.af. | 3389448 |
| #2 | ('Biodegradable polymer' or 'Bioabsorbable polymer' or 'Bioresorbable polymer' or 'Resorbable polymer' or 'Absorbable polymer' or 'Degradable polymer' or 'Biodegradable Stent' or 'Bioabsorbable stent' or 'Bioresorbable stent' or 'Resorbable stent' or 'Absorbable stent' or 'Degradable stent' or 'Bioresorbable scaffold' or 'Biodegradable scaffold' or 'Bioabsorbable scaffold' or 'Resorbable scaffold' or 'Absorbable scaffold' or 'Degradable scaffold' or BP-DES or Umirolimus or Biolimus).af. | 20876 |
| #3 | ('Permanent polymer' or 'Durable polymer' or 'Long-lasting polymer' or 'Enduring polymer' or 'Stable polymer' or 'Persistent polymer' or 'Permanent stent' or 'Durable stent' or 'Long-lasting stent' or 'Enduring stent' or 'Stable stent' or 'Persistent stent' or '2nd generation drug eluting stent' or 'Second generation drug eluting stent' or '2nd generation DP-DES' or 'Second generation DP-DES' or 'Second-generation pharmaceuticals' or '2nd generation pharmaceuticals' or 'Second-generation therapies' or '2nd generation therapies' or DP-DES or Zotarolimus).af. or Everolimus.sh. or Everolimus.af. | 45227 |
| #4 | Percutaneous coronary intervention.sh. or 'Percutaneous coronary intervention'.af. or angioplasty.sh. or angioplasty.af. or PCI.af. or 'Coronary angioplasty'.af. or 'Coronary stenting'.af. or 'Percutaneous transluminal coronary angioplasty'.af. or 'Percutaneous coronary angioplasty'.af. or 'Catheter-based coronary procedure'.af. or 'Interventional cardiology procedure'.af. or 'Percutaneous coronary procedure'.af. | 357377 |
| #5 | #1 AND #2 AND #3 AND #4 | 2444 |
| #6 | remove duplicates from 5 | 1873 |

**Scopus**

Search: 1/10/2024

|  | Search | Result |
| --- | --- | --- |
| #1 | TITLE-ABS-KEY ( "Long-term outcomes" OR "Long-term effects" OR "Clinical outcomes" OR "Long-term efficacy" OR "Patient outcomes" OR "Follow-up outcomes" OR "Prognosis" OR "Lasting effects" OR "Sustained outcomes" OR "Prolonged consequences" OR "Future impact" OR "Long-range results" OR "Final outcomes" ) | 1,986,821 |
| #2 | TITLE-ABS-KEY ( "Biodegradable polymer drug eluting stent" OR "Biodegradable polymer" OR "Bioabsorbable polymer" OR "Bioresorbable polymer" OR "Resorbable polymer" OR "Absorbable polymer" OR "Degradable polymer" OR "Biodegradable Stent" OR "Bioabsorbable stent" OR "Bioresorbable stent" OR "Resorbable stent" OR "Absorbable stent" OR "Degradable stent" OR "Bioresorbable scaffold" OR "Biodegradable scaffold" OR "Bioabsorbable scaffold" OR "Resorbable scaffold" OR "Absorbable scaffold" OR "Degradable scaffold" OR "BP-DES" OR "Umirolimus" OR "Biolimus" ) | 39,641 |
| #3 | TITLE-ABS-KEY ( "Durable polymer drug eluting stent (DP-DES)" OR "Permanent polymer" OR "Durable polymer" OR "Long-lasting polymer" OR "Enduring polymer" OR "Stable polymer" OR "Persistent polymer" OR "Permanent stent" OR "Durable stent" OR "Long-lasting stent" OR "Enduring stent" OR "Stable stent" OR "Persistent stent" OR "2nd generation drug eluting stent" OR "Second generation drug eluting stent" OR "2nd generation DP-DES" OR "Second generation DP-DES" OR "Second-generation pharmaceuticals" OR "2nd generation pharmaceuticals" OR "Second-generation therapies" OR "2nd generation therapies" OR "DP-DES" OR "Zotarolimus" OR "Everolimus" ) | 31,256 |
| #4 | TITLE-ABS-KEY ( "Percutaneous coronary intervention (PCI)" OR "Coronary angioplasty" OR "Angioplasty" OR "Coronary stenting" OR "Percutaneous Biodetransluminal coronary angioplasty (PTCA)" OR "PCI" OR "Percutaneous coronary angioplasty (PCA)" OR "Catheter-based coronary procedure" OR "Interventional cardiology procedure" OR "Percutaneous coronary procedure" ) | 164,348 |
| #5 | #1 AND #2 AND #3 AND #4 | 335 |
|  |  |  |

| **Long-term outcomes** | **Biodegradable polymer drug eluting stent** | **Durable polymer drug eluting stent (DP-DES)** | **Percutaneous coronary intervention (PCI)** |
| --- | --- | --- | --- |
| Long-term effects | Biodegradable polymer | Permanent polymer | Percutaneous coronary intervention (PCI) |
| Clinical outcomes | Bioabsorbable polymer | Durable polymer | Coronary angioplasty |
| Long-term efficacy | Bioresorbable polymer | Long-lasting polymer | Angioplasty |
| Patient outcomes | Resorbable polymer | Enduring polymer | Coronary stenting |
| Follow-up outcomes | Absorbable polymer | Stable polymer | Percutaneous transluminal coronary angioplasty (PTCA) |
| Prognosis | Degradable polymer | Persistent polymer | PCI |
| Lasting effects | Biodegradable Stent | Permanent stent | Percutaneous coronary angioplasty (PCA) |
| Sustained outcomes | Bioabsorbable stent | Durable stent | Catheter-based coronary procedure |
| Prolonged consequences | Bioresorbable stent | Long-lasting stent | Interventional cardiology procedure |
| Future impact | Resorbable stent | Enduring stent | Percutaneous coronary procedure |
| Long-rage results | Absorbable stent | Stable stent |  |
| Final outcomes | Degradable stent | Persistent stent |  |
|  | Bioresorbable scaffold | 2nd generation drug eluting stent |  |
|  | Biodegradable scaffold | Second generation drug eluting stent |  |
|  | Bioabsorbable scaffold | 2nd generation DP-DES |  |
|  | Resorbable scaffold | Second generation DP-DES |  |
|  | Absorbable scaffold | Second-generation pharmaceuticals |  |
|  | Degradable scaffold | 2nd generation pharmaceuticals |  |
|  | BP-DES | Second-generation therapies |  |
|  | Umirolimus | 2nd generation therapies |  |
|  | Biolimus | DP-DES |  |
|  |  | Zotarolimus |  |
|  |  | Everolimus |  |
