# Supplementary material for: Long-term outcomes of biodegradable versus 2nd generation durable polymer drug-eluting stents in PCI: Protocol for a systematic review and meta-analysis
Source: PLoS One. 2025 Mar 19;20(3):e0319946. doi: 10.1371/journal.pone.0319946 (PMC11922205; doi:10.1371/journal.pone.0319946)
Supplement: S3 File — Proforma used for GRADE assessment. (DOCX) [file pone.0319946.s003.docx]

**Rubric for GRADE assessment**

| **Domain** | **Level of limitation** | ***A priori* Domain Cutoffs** |
| --- | --- | --- |
| **Risk of Bias** | **“Serious”** = -1    **“Very Serious”** = -2 | The proportion of information from studies at high risk of bias is sufficient to affect the interpretation of results**.**  **“Serious”:** Most information is from studies at moderate risk of bias:  · >50% of trials received overall “some concerns” risk or  · 0-50% of trials received overall “High” risk of bias ratings  **“Very Serious”:** Most information is from studies at high risk of bias:  · >50 % of trials received overall “High” risk of bias ratings or  · >50% of trials received overall “some concerns” risk and 0-50% of trials received overall “High” risk of bias ratings |
| **Inconsistency** | **“Serious”** = -1    **“Very Serious”** = -2 | **“Serious”:** The examined RCT was judged to have **serious inconsistency** for the assessed outcome if I^2^ >50% and ≤75%  **“Very Serious”:** The examined RCT was judged to have **very serious inconsistency** for the assessed outcome if I^2^ >75%    Considerations for narrative outcomes:  - variation in effect estimates across studies  - sources of heterogeneity (study design, populations, interventions, comparators, outcomes measured)  - coherence of evidence |
| **Indirectness** | **“Serious”** = -1    **“Very Serious”** = -2 | The examined RCT was judged to have:  **“Serious” indirectness** if indirectness was present in any of the PICO components  **“Very serious indirectness”** if indirectness was present in two or more of the PICO components |
| **Imprecision** | **“Serious”**= -1    **“Very Serious”**= -2 | **“Serious”:** The examined RCT was judged to have **serious inconsistency** for the assessed outcome if:  · The 95% confidence interval (CI) crosses the line of no effect, or  · The event rate to sample size ratio is less than 0.5 and the CI is wide  **“Very Serious”: T**he examined RCT was judged to have **very serious inconsistency** for the assessed outcome if:  · The 95% CI crosses the line of no effect, and  · The event rate to sample size ratio is less than 0.5 and the CI is wide and/or  · There is clinical significant benefit or harm if the true effect of the intervention were to lie in the upper versus lower boundary of the CI |
| **Magnitude of Effect** | **“Large” = +1**    **“Very Large” = + 2** | The evidence will be upgraded by one level if 1 or 2 levels according to the criteria:  · effect is rapid  · effect is consistent across studies  · prediction interval does not cross the line of no effect  · previous trajectory of disease is reversed  · large magnitude of an effect is supported by indirect evidence |
